# Overexpression of NNT-AS1 Activates TGF-*β* Signaling to Decrease Tumor CD4 Lymphocyte Infiltration in Hepatocellular Carcinoma

**DOI:** 10.1155/2020/8216541

**Published:** 2020-12-23

**Authors:** Yakun Wang, Lei Yang, Xichen Dong, Xin Yang, Xinxue Zhang, Zhe Liu, Xin Zhao, Tao Wen

**Affiliations:** ^1^Medical Research Center, Beijing Chao-yang Hospital, Capital Medical University, China; ^2^Pathology Department, Beijing Cancer Hospital & Beijing Institute for Cancer Research, Beijing 100142, China; ^3^Hepatobiliary Surgery Department, Beijing Chao-yang Hospital, Capital Medical University, Beijing 100020, China

## Abstract

Nicotinamide nucleotide transhydrogenase-antisense RNA1 (NNT-AS1) is a long noncoding RNA (lncRNA) that has been shown to be overexpressed in hepatocellular carcinoma (HCC). However, the molecular mechanism involving NNT-AS1 in HCC remains to be extensively investigated. The activation of TGF-*β* signaling inhibits tumor-infiltrating lymphocytes (TILs) and results in tumor immune evasion. We thus planned to explore the mechanism by which NNT-AS1 activates the TGF-*β* signaling pathway and inhibits TILs in HCC. High levels of NNT-AS1 were detected in HCC tissues by both RNAscope and real-time quantitative PCR (RT-qPCR) assays. The levels of proteins involved in TGF-*β* signaling and those of CD4 T lymphocytes were quantified by immunohistochemistry (IHC). HCC cell lines (HepG2 and Huh7) were used to explore the effects of NNT-AS1 on TGF-*β* signaling activation. In these analyses, RNAscope detection demonstrated that NNT-AS1 levels were significantly increased in HCC cancer tissues (*P* = 0.0001). In addition, the elevated NNT-AS1 levels in cancer tissue were further confirmed by RT-qPCR analysis of HCC cancer tissues (*n* = 64) and normal tissues (*n* = 26) (*P* = 0.0003). Importantly, the overall survival time of HCC patients who exhibited higher levels of NNT-AS1 expression was significantly shorter than that of HCC patients who had lower levels of NNT-AS1 expression (*P* = 0.0402). Further mechanistic investigation indicated that NNT-AS1 inhibition significantly decreased the levels of TGF-*β*, TGFBR1, and SMAD5 in HCC cells. In HCC tissues, IHC detection showed that relatively high NNT-AS1 levels were associated with a reduction in infiltrated CD4 lymphocyte numbers. In conclusion, this research identifies a novel mechanism by which NNT-AS1 impairs CD4 T cell infiltration via activation of the TGF-*β* signaling pathway in HCC.

## 1. Introduction

Hepatocellular carcinoma (HCC) is a malignant disease with high mortality worldwide [1]. Immune checkpoint blockade (ICB) increasingly being applied as a treatment for advanced HCC [2]. For instance, nivolumab (a PD-1 inhibitor) has been approved as a second line for HCC treatment [3, 4]. Increasing tumor-infiltrating lymphocyte (TIL) levels is the most important way to improve ICB treatment [5]. However, elucidation of the molecular mechanisms governing the TILs in HCC to improve ICB clinical effectiveness is still needed.

lncRNAs have recently been identified as important drivers or inhibitors of carcinogenesis [6]. For example, lncRNA-ATB and SNHG6-003 were demonstrated to promote cell proliferation and metastasis in HCC [7, 8]. p18 is a cancer suppressor, whose expression can be downregulated by lncRNA/HULC to increase HCC cell proliferation [9]. HCC stem cell proliferation is promoted by lncRNA/CUDR, which results in a poor prognosis in HCC patients [10]. In addition, NNT-AS1 was identified as a driver gene in cervical cancer [11], gastric cancer [12], and osteosarcoma [13]. Mechanistically, NNT-AS1 was identified as a ceRNA involved in the epithelial-to-mesenchymal transition (EMT) process, which was activated by upregulating ZEB1 expression and inhibiting miR-142-3p [14]. In the tumor microenvironment (TME), cancer-cell-derived TGF-*β* induces the formation of an immunosuppressive TME [15]. Tumor immune evasion can be mediated by TGF-*β* signaling that increases IDO and CCL22 expressions in dendritic cells (DCs) [16]. CD8 CD103 TILs in tumor beds can be inhibited by TGF-*β* and develop a tolerogenic phenotype [17]. Thus, a new ICB treatment strategy employing antibodies that could block both TGF-*β* and PD1 was developed. Those antibodies have been validated in a preclinical model [18]. Our previous investigations demonstrated that the TGF-*β* signaling pathway was hyperactive in the TME of colorectal cancer and enhanced angiogenesis [19, 20].

In the present study, we aimed to investigate NNT-AS1 expression in HCC by applying RNAscope *in situ* hybridization. The associations of NNT-AS1 with clinical pathology were also extensively analyzed in HCC patients. Our mechanistic analyses uncovered the novel mechanism by which NNT-AS1 activates TGF-*β* signaling and the associated effects on TILs in HCC.

## 2. Materials and Methods

### 2.1. HCC Samples

The tissue samples used to construct tissue microarrays (TMAs) were collected from 16 HCC patients at Beijing Chao-yang Hospital and Beijing Cancer Hospital. All enrolled patients signed informed consent forms. Our study was approved by the Beijing Chao-yang Hospital Ethics Committee. A tissue cDNA array (cDNA-HLivH090Su01) that included 90 samples was obtained from OUTDO (Shanghai OUTDO Biotech Co., Ltd., Shanghai, China).

### 2.2. RNAscope *In Situ* Hybridization Assay

The methods for RNAscope and standards for quantitative calculation were described in our previous studies [21, 22]. An RNAscope assay kit (RNAscope® 2.5 HD Assay-RED, Advanced Cell Diagnostics, Hayward, CA, USA, Cat No. 322310) and a probe targeting NNT-AS1 (ACD, Cat No. 17268B) were applied to detect NNT-AS1 in an HCC TMA. Briefly, the TMA slides were deparaffinized and then subjected to epitope retrieval. After protease and H_2_O_2_ blocking, the NNT-AS1 probes were applied for hybridization and amplification. The signals per cell were manually counted.

### 2.3. TCGA Data Analysis

The LIHC dataset was downloaded from The Cancer Genome Atlas (TCGA) data portal (http://www.cbioportal.org/). The LIHC dataset contained the transcripts per kilobase of exon model per million mapped read (TPM) values and overall survival (OS) information. These data were used to determine the difference in NNT-AS1 levels between HCC tissues and normal tissues by standardizing the mRNA expression profiles generated by RNA sequencing. The TPM quartile value was used as the cutoff value to separate LIHC patients with low or high levels of NNT-AS1 for OS analysis.

### 2.4. Cell Lines and Culture

HCC cell lines, which included Huh7 and HepG2, and the normal liver cell line HL-7702 were obtained from the Cell Center of the Institute of Basic Medicine, Peking Union Medical College. Huh7 and HepG2 cells were cultured in DMEM, while HL-7702 cells were cultured in RPMI 1640 medium. All media were supplemented with 10% FBS and 1% penicillin/streptomycin. Cells were incubated at 37°C in 5% CO_2_.

### 2.5. siRNA Transfection

siRNAs were synthesized by SyngenTech (Beijing, China). siRNA transfections into cells were carried out with Lipofectamine 3000 (Life Technologies) according to the manufacturer's recommendations. The sequences for siRNAs were as follows:

NNT-AS1: siNTT1, 5′-GCCAGUCCUUGUCAAUCAATT-3′; siNTT2, 5′-GCCUUUCUAGGCUGUACAATT-3′; siNTT3, 5′-GGAGACAGAUGGAUCAUUUTT-3′; siNTT4, 5′-GAAAAGAAAAAGAAGCUUAtt-3′; siTGF-*β*, 5′-ACAACGAAAUCUAUGACAATT-3′; siTGFBR1, 5′-GAACAGAAGUUAAGGCCAATT-3′; siSMAD5, 5′-GAGCUAAAGCCGUUGGAUATT-3′; and control siRNA, 5′-UUCUCCGAACGUGUCACGUTT-3′.

### 2.6. RNA Extraction, Reverse Transcription, and Real-Time Quantitative Reverse-Transcriptase Polymerase Chain Reaction (RT-qPCR)

After extracting total RNA (TRIzol, Invitrogen, Carlsbad, CA, USA), 1 *μ*g of total RNA was reverse transcribed to produce complementary DNA (cDNA) with the First-Strand cDNA Synthesis Kit (Transgene, Beijing, China). Then, the template cDNAs were evaluated by RT-qPCR with SYBR mix (Transgene, Beijing, China) on the Applied Biosystems 7500 Real-Time PCR System (Life Technologies, Gaithersburg, MD, USA). The procedures were as follows: 95°C predenaturation for 5 min, followed by 40 cycles of 95°C for 15 s, and annealing at 60°C for 30 s. The primer sequences were as follows:

NNT-AS1: F, 5′-CAAAAGGCGACCTCACGAAAT-3′; R, 5′-TTGATTGACAAGGACTGGCG-3′; TGF-*β*: F, 5′-ATGGAGAGAGGACTGCGGAT-3′; R, 5′-TAGTGTTCCCCACTGGTCCC-3′; TGFBR1: F, 5′-TCCAACTACTGTAAAGTCATCACC-3′; R, 5′-GGGTCCTCTTCATTTGGCAC-3′; SMAD5: F, 5′-AATCTGCCTCTGACTTGACCC-3′; R, 5′-CGGAGACCTTCCTGTAACTCAA-3′; and 18S: F, 5′-AAACGGCTACCACATCCA-3′; R, 5′-CACCAGACTTGCCCCTCCA-3′.

### 2.7. Western Blot Analysis

Cells were lysed with 50 *μ*L of RIPA buffer (Solarbio, Shanghai, China) containing protease inhibitors (Invitrogen, Carlsbad, CA, USA) and phosphatase inhibitors. The lysates were electrophoresed on a 10% SDS-PAGE gel, and then the proteins were transferred to a polyvinylidene fluoride (PVDF) membrane. After blocking with 5% skim milk, the membrane was incubated with primary antibodies against TGF-*β* (CST, Danvers MA, 1 : 200 dilution), TGFBR1 (Abcam, 1 : 400 dilution), SMAD5 (Abcam, 1 : 200 dilution), and GAPDH (CST) overnight at 4°C. After washing with TBST, an HRP-labeled secondary antibody (Zhongshan Golden Bridge, China) was applied and incubated at room temperature for 1 hour to detect the primary antibodies. Protein bands were visualized using Super Enhanced Chemiluminescence Detection regents (Applygen Technologies, Beijing, China).

### 2.8. Immunohistochemical (IHC) Analysis

The TMAs were heated at 65°C for 2 hours. Then, the TMAs were dewaxed with xylene and gradient ethanol and rehydrated. The TMAs were immersed in an EDTA solution (pH = 8.0) for antigen retrieval with boiling in a microwave for 15 min. Goat serum (2%) was used to block the TMAs. Subsequently, the TMAs were incubated with primary antibodies (anti-TGF-*β*, dilution 1 : 100; anti-TGFBR1, dilution 1 : 100; anti-SMAD5, dilution 1 : 100; and anti-CD4, dilution 1 : 100, Abcam) overnight at 4°C.

### 2.9. Statistical Analysis

*Chi*-squared and *Wilcoxon rank-sum* tests were applied where appropriate to compare the differential expression of NNT-AS1 between cancer and normal tissues and the associations of NNT-AS1 with clinicopathological parameters (SPSS 11.0 statistical software or GraphPad Prism 7). Kaplan-Meier analysis and the Cox proportional hazards regression model were used to analyze the prognostic relevance of NNT-AS1. Correlational analyses (Pearson correlation) were performed using the GraphPad Prism 7 software or R software (3.5.2). Survival curves were plotted with the GraphPad Prism 7 software. The data were shown as the mean ± SD. Unpaired *t*-test was used to compare the mean values (two-tailed). All statistical analyses were conducted at a significance level of *P* < 0.05.

## 3. Results

### 3.1. RNAscope Detection Demonstrated Overexpression of NNT-AS1 in HCC

**Figure 1:**
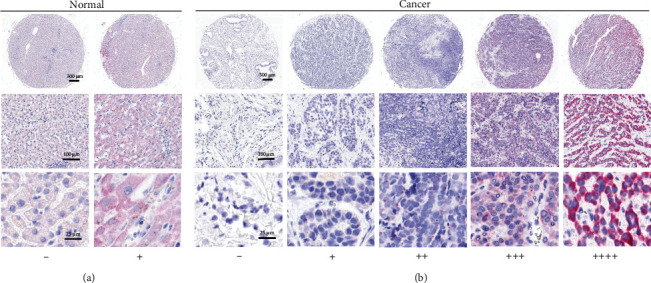
Representative picture images of RNAscope staining for NNT-AS1 in (a) HCC adjacent normal tissues and (b) HCC cancer tissues. “-” (0-1 dots/10 cells), “+” (1-3 dots/cell), “++” (4-10 dots/cell), “+++” (>10 dots/cell with dots in clusters <10%), and “++++” (>10 dots/cell with dots in clusters >10%).

First, we investigated the differential expression of NNT-AS1 in HCC. An RNAscope assay was carried out with TMA slides to visualize NNT-AS1. RNAscope detection demonstrated that NNT-AS1 was overexpressed and located in the cytoplasm of HCC cells, while there was no NNT-AS1 signal in stromal cells (Figures 1(a) and 1(b)). To quantitively compare the differential expression of NNT-AS1, we classified the RNAscope scores as follows: “-” and “+” represented low levels and “++,” “+++,” and “++++” represented high levels of NNT-AS1. Then, the differential expression of NNT-AS1 was compared. Our analyses demonstrated that 10 (62.5%) of 16 HCC tissues had high levels of NNT-AS1. Meanwhile, low NNT-AS1 expression was observed in paired adjacent normal tissues (*P* = 0.001) (Table 1). Overall, our RNAscope analysis demonstrated that NNT-AS1 was overexpressed in HCC tissues.

### 3.2. High Levels of NNT-AS1 in HCC Tissues Were Validated by RT-qPCR

**Figure 2:**
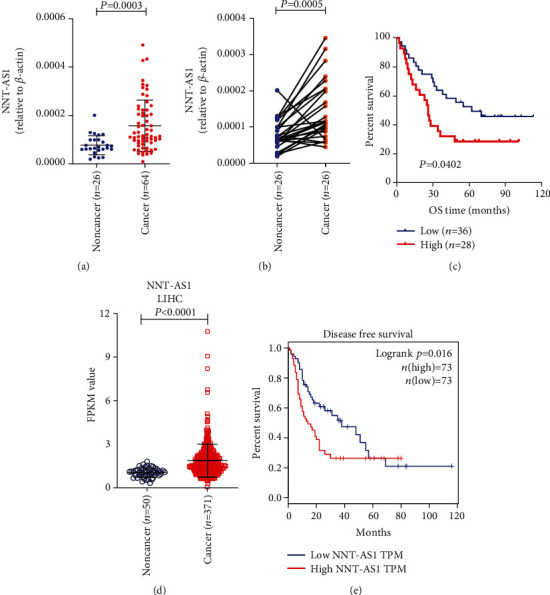
(a) RT-qPCR detection of the expression of NNT-AS1 in cancer and adjacent nontumor tissues from 64 patients with HCC. (b) The expression of NNT-AS1 in cancer tissues and adjacent normal tissues in 26 paired samples from HCC patients was detected by RT-qPCR. (c) Kaplan-Meier analysis of the OS of 64 patients with HCC. (d) NNT-AS1 overexpression was validated in TCGA LIHC dataset. (e) Elevated expression of NNT-AS1 was found to be associated with a shortened overall survival (OS) time in LIHC.

We further detected the overexpression of NNT-AS1 by RT-qPCR in a larger cohort that included 64 HCC patients. This analysis confirmed that NNT-AS1 expression was significantly higher in HCC tissues (*n* = 64) than in normal tissues (*n* = 26) (*P* = 0.0003) (Figure 2(a)). We also compared the differential levels of NNT-AS1 in 26 paired tissue samples that included cancer and normal tissues from the same HCC patient. The levels of NNT-AS1 were found to be increased in 76.92% (20/26) of the paired HCC tissue samples. Statistical analysis indicated that the levels of NNT-AS1 were significantly higher in the cancer tissue samples than in the paired normal tissue samples (*P* = 0.0005) (Figure 2(b)). Both RT-qPCR and RNAscope provided solid evidence that NNT-AS1 was overexpressed in HCC cells.

### 3.3. NNT-AS1 Is a Prognostic Factor in HCC

Next, we wanted to determine the clinicopathological and prognostic relevance of NNT-AS1 in HCC. Both Kaplan-Meier and *Cox* regression analyses were applied to evaluate the prognostic relevance of NNT-AS1 in HCC. cDNA samples from 64 enrolled HCC patients who were followed for 2-113 months (44.25 ± 31.45) were evaluated by RT-qPCR. At the endpoint of the follow-up period, 39 (60.94%) of the HCC patients had died. Kaplan-Meier methods were used to plot OS curves based on the level of NNT-AS1 (high vs. low), and the curves indicated that NNT-AS1 was a prognostic factor in HCC patients (*P* = 0.0402) (Figure 2(c)). *Cox* univariate analyses demonstrated that tumor size (≤5 cm vs. >5 cm, *P* = 0.028), TNM stage (I vs. II/III, *P* = 0.036), and NNT-AS1 level (higher vs. lower, *P* = 0.044) were significantly associated with the OS of HCC patients (Table 2).

We next explored the prognostic relevance of NNT-AS1 by analyzing TCGA data (LIHC). The LIHC dataset contains the RNA sequencing results of 369 cancerous tissue samples and 50 normal tissue samples. The normalized TPM values of NNT-AS1 for each tissue sample were calculated to validate the differential expression of NNT-AS1 between cancer and normal tissues. In accordance with our RNAscope and RT-qPCR results, analysis of the LIHC data confirmed the overexpression of NNT-AS1 in HCC (*P* < 0.0001) (Figure 2(d)). A prognostic investigation was performed with an LIHC dataset that included 146 HCC patients. The level of NNT-AS1 was found to be associated with OS time (*P* = 0.016) (Figure 2(e)). These results indicated that NNT-AS1 was a driver gene that could promote HCC progression and result in a poor prognosis.

### 3.4. Bioinformatic Analyses Explored whether NNT-AS1 Is Associated with Immune Reactions and TGF-*β* Signaling

**Figure 3:**
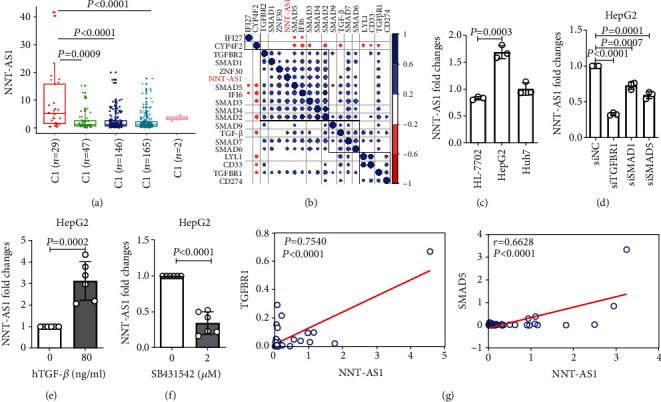
Six immune subtypes of cancer, C1 (wound healing), C2 (IFN-*γ* dominant), C3 (inflammation), C4 (lymphocyte depletion), C5 (immune silencing), and C6 (TGF-*β* dominance). There were no samples with the C5 subtype in the LIHC data. (a) Six immune subtypes of cancer, C1 (wound healing), C2 (IFN-*γ* dominant), C3 (inflammation), C4 (lymphocyte depletion), C5 (immune silencing), and C6 (TGF-*β* dominance). There were no samples with the C5 subtype in the LIHC data. The levels of NNT-AS1 in different HCC immune subtypes. (b) *Pearson* correlation analysis of the levels of NNT-AS1 and genes involved in TGF-*β* signaling and other genes involved in interferon signaling and the immune reaction. (c) RT-qPCR determination of the NNT-AS1 levels in three cell lines (HL-7702, HepG2, and Huh7, repeat three times). (d) NNT-AS1 expression was confirmed by RT-qPCR after transfection of siRNA targeting TGF-*β*, TGFBR1, or SMAD5 into HepG2 cells (repeat three times). (e, f) Human recombinant TGF-*β* activated the TGF-*β* signaling pathway and increased the expression of NNT-AS1. SB431542 inhibited the TGF-*β* signaling pathway and decreased the expression of NNT-AS1 (repeat 6 times). (g) The correlations of NNT-AS1 with SMAD5 and TGFBR1 in 15 HCC patients were analyzed by RT-qPCR.

A previous study had summarized six cancer immune subtypes based on TCGA datasets [23]. We applied integrative bioinformatic investigation to compare the levels of NNT-AS1 among LIHC samples representing different immune subtypes. The level of NNT-AS1 was found to be significantly increased in HCC patients with the C1 (wound healing) type. This cancer immune subtype was shown to have rare TILs and shorter OS time (Figure 3(a)). We thus speculated that NNT-AS1 may regulate the immune response in HCC.

Bioinformatic analyses of TCGA data were used to examine the relationships between NNT-AS1 and genes involved in TGF-*β* signaling (Figure 3(b)). Our analyses proved that the level of NNT-AS1 was significantly associated with the mRNA levels of *TGF-β*, *TGFBR1*, *SMAD1*, *SMAD2*, *SMAD3*, *SMAD4*, *SMAD5*, *SMAD6*, and *SMAD7*. Interferon signaling was the dominant pathway in the C2 (interferon *γ*) immune type (Figure 3(a)). The level of TGF-*β* was negatively associated with genes involved in interferon signaling (such as IFI27, IFI26, and LYL1). CYP4F2, a liver enzyme that metabolizes fatty acids, vitamin D, and carcinogens which is expressed at relatively low levels in HCC [24], was found to be negatively associated with TGF-*β* signaling (Figure 3(b)). CD33 is a marker of myeloid-derived suppressor cells (MDSCs), and its overexpression is associated with relatively poor survival rates in HCC [25]. Here, we identified that CD33 was positively associated with TGF-*β* signaling (Figure 3(b)). Our bioinformatic analyses based on LIHC datasets indicated that NNT-AS1 plays a positive role in activating TGF-*β* signaling and a negative role in regulating antitumor immune reactions.

Next, we evaluated cell lines to determine the underlying mechanism by which NNT-AS1 regulates TGF-*β*. The level of NNT-AS1 was higher in HepG2 cells than in HL-7720 cells (a normal liver cell line) (Figure 3(c)). We thus selected HepG2 cells to investigate whether downregulation of TGF-*β* signaling decreases NNT-AS1 expression. The mRNA levels of NNT-AS1 were significantly decreased after transfecting siRNAs against *TGFBR1*, SMAD1, or *SMAD5* (Figure 3(d)). However, there were no effects on the expression of NNT-AS1 after transfecting siRNAs targeting *TGF-β* or *SMAD9*. These results are consistent with the above bioinformatic analyses.

Consistent with these findings, we activated the TGF-*β* signaling pathway by adding human recombinant TGF-*β* to the medium of HepG2 cells, which significantly increased NNT-AS1 levels (Figure 3(e)). Furthermore, we inhibited the TGF-*β* signaling pathway by adding SB431542, an inhibitor that can block the TGF-*β* receptor, to the medium and found significantly decreased NNT-AS1 levels (Figure 3(f)). According to the above analyses, both endogenous inhibition of TGF-*β* signaling by siRNA transfection and exogenous treatment with chemokines (hTGF-*β* and SB431542) that regulate TGF-*β* signaling significantly changed the expression of NNT-AS1.

To confirm our findings, we next detected the mRNA levels of NNT-AS1, *TGF-β*, *SMAD5*, and *TGFBR1* in HCC tissue samples by RT-qPCR. The levels of NNT-AS1 were demonstrated to be positively associated with the mRNA levels of SMAD5 and TGFBR1. However, there was no significant correlation between NNT-AS1 and TGF-*β* (Figure 3(g)). This lack of association might have resulted from the relatively small cohort evaluated in this analysis.

### 3.5. NNT-AS1 Impacts TGF-*β* Signaling by Regulating TGF-*β*, TGFBR1, and SMAD5 in HCC Cells

**Figure 4:**
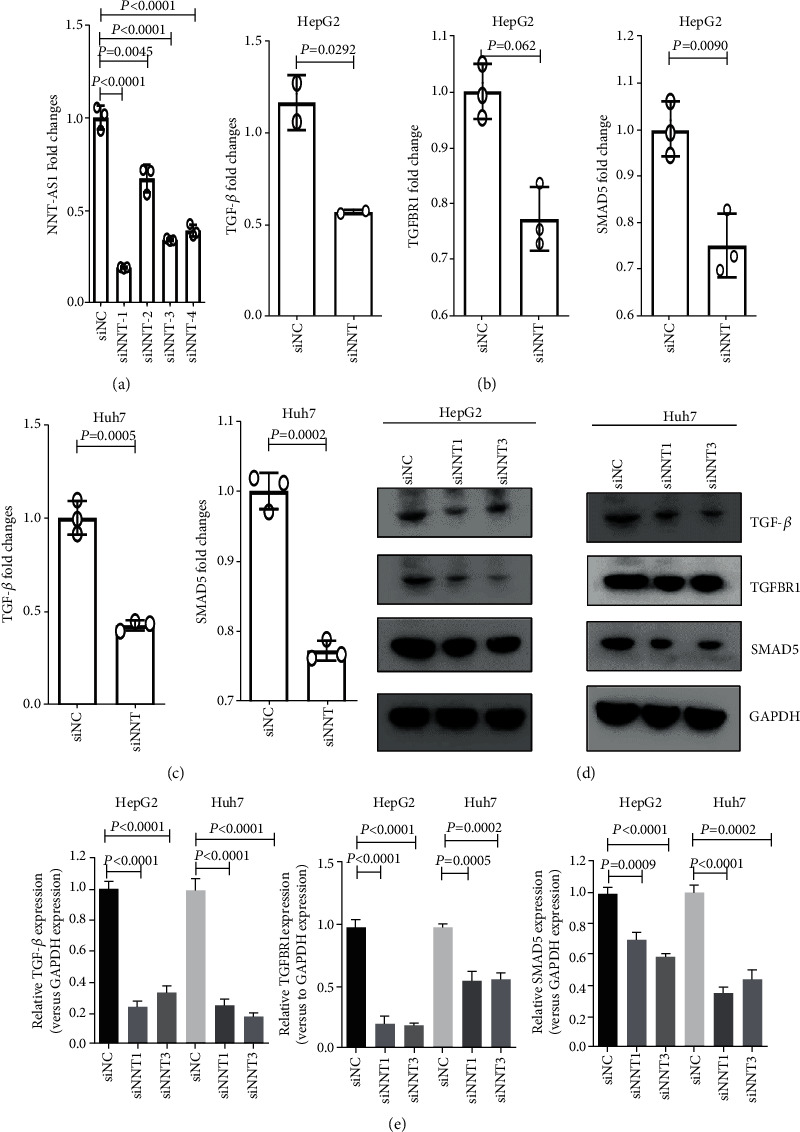
NNT-AS1 expression levels in cells transiently transfected with NNT-AS1-specific siRNAs (siNNT) or control siRNA (siNC). (a) NNT-AS1 expression was determined by RT-qPCR after siRNA transfection of HepG2 cells (repeat three times). (b) RT-qPCR assays determined the mRNA levels of *TGF-β*, *TGFBR1*, and *SMAD5* in HepG2 (repeat three times). (c) RT-qPCR assays determined the mRNA levels of *TGF-β* and *SMAD5* in Huh7 (repeat three times). (d) Representative images of Western blotting that were used to determine the protein levels of TGF-*β*, TGFBR1, and SMAD5 in HepG2 and Huh7 cells. (e) The ImageJ software was used for semiquantitative analysis of the relative levels of TGF-*β*, TGFBR1, and SMAD5 based on the results from three Western blotting assays.

We explored whether NNT-AS1 levels are associated with TGF-*β* signaling. We next investigated the roles of NNT-AS1 in regulating genes involved in TGF-*β* signaling. To downregulate NNT-AS1 expression, we synthesized four siRNAs targeting the mRNA sequence of NNT-AS1 and transfected them into HepG2 cells. As shown in Figure 4(a), siNNT1 and siNNT3 exhibited more effective inhibitory effects producing NNT-AS1 downregulation (Figure 4(a)). We detected the mRNA levels of *TGF-β*, *TGFBR1*, and *SMAD5* by RT-qPCR. The levels of *TGF-β*, *TGFBR1*, and *SMAD5* were significantly decreased after downregulating NNT-AS1 expression in HepG2 cells (Figure 4(b)). Huh7 cells were demonstrated to have lower levels of NNT-AS1 than HepG2 cells (Figure 3(c)). The downregulation of NNT-AS1 expression significantly decreased the levels of *TGF-β* and *SMAD5* in Huh7 cells but had no effect on *TGFBR1* expression (Figure 4(c)). These results indicated that endogenous NNT-AS1 expression affected the impact of siRNA transfection on TGF-*β* signaling regulation. Moreover, we found decreased protein levels of TGF-*β*, TGFBR1, and SMAD5 after transient transfection of siNNT1 or siNTT3 into HepG2 and Huh7 cells (Figures 4(d) and 4(e)). These results suggest that NNT-AS1 is involved in TGF-*β* signaling by affecting the transcription of TGF-*β*, TGFBR1, and SMAD5.

### 3.6. NNT-AS1 Is Positively Correlated with the TGF-*β* Signaling Pathway but Negatively Correlated with T Cell Infiltration in HCC

**Figure 5:**
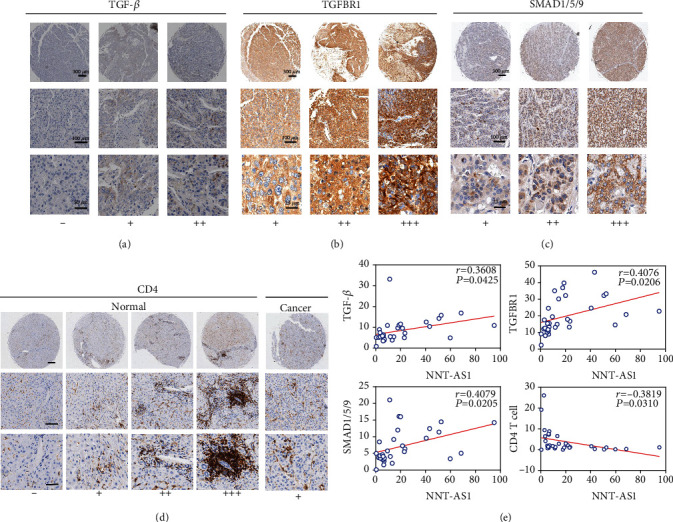
IHC analyses were used to determine the levels of (a) TGF-*β*, (b) TGFBR1, (c) SMAD1/5/9, and (d) CD4 T cells in continuous TMA slides. *Pearson* correlation analyses were used to determine the correlation between NNT-AS1 levels and TGF-*β*, TGFBR1, SMAD1/5/9, and CD4 T cell levels (e).

TGF-*β* signaling has been proven to inhibit immune reactions and thus favor tumor immune evasion. In the present study, we investigated whether there are associations among NNT-AS1, TGF-*β* signaling, and TILs in HCC tissue. IHC analysis was performed with HCC TMAs to determine the levels of TGF-*β*, TGFBR1, SMAD1/5/9, CD4, and CD8 in continuous TMA slides. Through semiquantitative scoring of IHC results, we evaluated the relationships of NNT-AS1 expression with the levels of TGF-*β*, TGFBR1, SMAD1/5/9, CD4 T cells, and CD8 T cells. In Figure 5, we show representative images depicting the levels of TGF-*β* (Figure 5(a)), TGFBR1 (Figure 5(b)), SMAD1/5/9 (Figure 5(c)), and CD4 T cells (Figure 5(d)) in HCC tissue. The levels of TGF-*β*, TGFBR1, and SMAD1/5/9 were significantly increased in HCC tissue (Table 3). Conversely, the levels of CD4 T cells were significantly reduced in cancer tissue (Figure 5(d)) (Table 3). Fewer infiltrated CD8 T cells were detected in HCC tissue (data not shown), which indicated that cytotoxic T cell effects were inhibited in HCC.

We evaluated the correlations among the levels of TGF-*β*, TGFBR1, SMAD1/5/9, and CD4 T cells (Figure 5(e)). The *Pearson* correlation test demonstrated that the levels of NNT-AS1 were positively related to the expression of TGF-*β*, TGFBR1, and SMAD1/5/9. Importantly, there was a negative association between the levels of NNT-AS1 and the levels of CD4 T cells, suggesting that NNT-AS1 inhibited CD4 T cell infiltration in HCC (Figure 5(e)). Collectively, these results indicate that NNT-AS1 activates the TGF-*β* signaling pathway and thus inhibits CD4 T cell tumor infiltration in HCC.

## 4. Discussion

Next generation sequence (NGS) has been widely used to explore novel differential lncRNA in cancer [26]. These lncRNAs participate in multiple tumorigenic processes and promote cancer cell proliferation and metastasis^,^ [27]. However, there is still an urgent need to examine most lncRNAs to determine their effects. Our present research identified a novel mechanism affecting TILs in which NNT-AS1 increased the activity of TGF-*β* signaling and thus inhibited CD4 T cell infiltration. This mechanism suggests that a strategy involving inhibition of NNT-AS1 might improve ICB effectiveness in clinical practice.

NNT-AS1 overexpression has been reported in several studies [28], but some studies have found that NNT-AS1 expression is decreased in cancer [29]. This discrepancy might result from using cancer samples containing mixed nonneoplastic cells. RNAscope was developed to evaluate RNA molecules *in situ* in cancer tissues, which allows us to compare lncRNAs between cancer cells and nonmalignant cells [30]. Here, we employed RNAscope to determine whether NNT-AS1 expression is unregulated in HCC tissues. Then, RT-qPCR was further applied to validate the overexpression of NNT-AS1 in HCC tissues. These findings more precisely demonstrated that NNT-AS1 expression is increased in cancer that NNT-AS1 acts as a driver in HCC.

Our present study firmly indicates that NNT-AS1 enhanced the activation of TGF-*β* signaling. The SMAD2/3 and SMAD1/5/9 pathways are the major activated downstream signaling pathways after TGF-*β* binds to TGFBR1 [31]. SMAD5 is the major component of the SMAD1/5/9 complex that is often activated by BMP in several cancer types [32]. Taking these results together with previous work, we proposed that NNT-AS1 successively activates the TGF-*β* and SMAD1/5/9 pathways. Previous studies have explored the roles of NNT-AS1 as a driver in carcinogenesis and cancer progression. For example, NNT-AS1 promotes cancer cell proliferation by sponging miR-203 in cholangiocarcinoma [33]. NNT-AS1/miR-3666/E2F2 forms a signaling axis that regulates lung cell proliferation [34]. NNT-AS1 also proved to sponge miR-363 to promote gastric cancer proliferation and invasion [12]. Our mechanistic investigations of NNT-AS1-mediated regulation of the TGF-*β* pathway are novel. TGF-*β* acts as an immunosuppressive cytokine that effects on both immune cell differentiation and immune cell proliferation [35]. Tumor-derived TGF-*β* proteins can decrease the proliferation of T lymphocytes [36] and thymocytes [37]. In glioblastoma, TGF-*β*2 is a T cell suppressor and is associated with immunosuppression [38]. Overall, our present study and other reports have indicated that NNT-AS1 overexpression promotes HCC progression by enhancing cancer cells' proliferation and metastasis and inhibiting TILs via TGF-*β* signaling activation.

CD4 T cells play important antitumor roles and are responsible for T cell receptor-mediated activation of the adaptive immune system via recognition of MHC class-II antigens [39]. CD4 T cells exert antitumor effects by secreting IL-33 [40]. TGF-*β* drives immune evasion by inhibiting CD4 T cell infiltration [41]. In addition, TGF-*β* combined with IL-2 was proven to promote CD4 T cell differentiation into Treg cells [42, 43]. All of the above reports clearly indicate that TGF-*β* signaling contributes to inhibiting antitumor immune reactions. In our study, we found that the levels of tumor-infiltrating CD4 T lymphocytes were negatively associated with the levels of NNT-AS1. Therefore, we proposed a novel strategy to improve ICB treatment in HCC through expanding the CD4 TIL population by inhibiting NNT-AS1 to inactivate TGF-*β* signaling.

In summary, we demonstrated that NNT-AS1 is a prognostic factor in HCC tissues that can be detected by RNAscope methods and developed a potential avenue for improving clinical treatment. Our research identified the novel mechanism by which NNT-AS1 enhanced the TGF-*β* signaling pathway and further decreased CD4 lymphocyte infiltration in HCC. However, there were several limitations to our study. For example, this was a retrospective study with relatively small sample sizes. Future studies should be performed to address the clinical applicability of targeting NNT-AS1.

## Figures and Tables

**Table 1:** RNAscope determination of the levels of NNT-AS1 in hepatocellular carcinoma.

Score	NNT-AS1 expression	
	Normal (*n* = 16)	Cancer (*n* = 16)
-	13 (81.25%)	2 (12.50%)
+	3 (18.75%)	4 (25.00%)
++	0	6 (37.50%)
+++	0	2 (12.50%)
++++	0	2 (12.50%)
*P* value	0.001	

**Table 2:** *Cox*'s proportional hazards model analyses of the association between NNT-AS1 and overall survival.

Variables	Univariate analysis		Multivariate analysis	
	HR (95% CI)	*P* value	HR (95% CI)	*P* value
NNT-AS1 (low vs. high)	1.918 (1.017-3.619)	0.044	1.701 (0.694-4.167)	0.245
Sex (female vs. male)	1.335 (0.321-5.546)	0.691		
Age (≤60 vs. >60)	0.734 (0.348-1.549)	0.471		
Tumor size (≤5 cm vs. >5 cm)	2.071 (1.082-3.962)	0.028	1.932 (0.738-5.056)	0.180
Tumor number (single vs. multiple)	1.198 (0.367-3.915)	0.765		
Pathological grade (H, M vs. L)	2.053 (0.838-5.029)	0.116	1.540 (0.568-4.178)	0.396
TNM stage (I vs. II, III)	2.113 (1.049-4.255)	0.036	2.806 (1.052-7.481)	0.039

CI: confidence interval; HR: hazard ratio.

**Table 3:** IHC staining for TGF-*β*, TGFBR1, SMAD1/5/9, and CD4 in HCC tissue samples.

Score	TGF-*β*		TGFBR1		SMAD1/5/9		CD4	
	Normal (*n* = 16)	Cancer (*n* = 16)	Normal (*n* = 16)	Cancer (*n* = 16)	Normal (*n* = 16)	Cancer (*n* = 16)	Normal (*n* = 16)	Cancer (*n* = 16)
-	5 (31.25%)	0	0	1 (6.25%)	9 (56.25%)	1 (6.25%)	1 (6.25%)	3 (18.75%)
+	9 (56.25%)	11 (68.75%)	13 (81.25%)	7 (43.75%)	7 (43.75%)	2 (12.50%)	2 (12.50%)	5 (31.25%)
++	2 (12.50%)	4 (12.50%)	2 (12.50%)	6 (37.50%)	0	11 (68.75%)	6 (37.50%)	8 (50.00%)
+++	0	1 (6.26%)	1 (6.26%)	2 (12.50%)	0	2 (12.50%)	7 (43.75%)	0
*P* value	0.046		0.005		0.008		0.002	

## Data Availability

The data used to support the findings of this study are included within the supplementary information file(s).
